# Predicting Species-Resolved Macronutrient Acquisition during Succession in a Model Phototrophic Biofilm Using an Integrated ‘Omics Approach

**DOI:** 10.3389/fmicb.2017.01020

**Published:** 2017-06-13

**Authors:** Stephen R. Lindemann, Jennifer M. Mobberley, Jessica K. Cole, L. M. Markillie, Ronald C. Taylor, Eric Huang, William B. Chrisler, H. S. Wiley, Mary S. Lipton, William C. Nelson, James K. Fredrickson, Margaret F. Romine

**Affiliations:** ^1^Biological Sciences Division, Pacific Northwest National Laboratory, RichlandWA, United States; ^2^Whistler Center for Carbohydrate Research, Department of Food Science, Purdue University, West LafayetteIN, United States; ^3^Department of Nutrition Science, Purdue University, West LafayetteIN, United States; ^4^Environmental Molecular Sciences Laboratory, Pacific Northwest National Laboratory, RichlandWA, United States

**Keywords:** carbon fixation, nitrate reduction, phosphate transport, sulfate reduction, metagenomics, metatranscriptomics, metaproteomics, periphyton

## Abstract

The principles governing acquisition and interspecies exchange of nutrients in microbial communities and how those exchanges impact community productivity are poorly understood. Here, we examine energy and macronutrient acquisition in unicyanobacterial consortia for which species-resolved genome information exists for all members, allowing us to use multi-omic approaches to predict species’ abilities to acquire resources and examine expression of resource-acquisition genes during succession. Metabolic reconstruction indicated that a majority of heterotrophic community members lacked the genes required to directly acquire the inorganic nutrients provided in culture medium, suggesting high metabolic interdependency. The sole primary producer in consortium UCC-O, cyanobacterium *Phormidium* sp. OSCR, displayed declining expression of energy harvest, carbon fixation, and nitrate and sulfate reduction proteins but sharply increasing phosphate transporter expression over 28 days. Most heterotrophic members likewise exhibited signs of phosphorus starvation during succession. Though similar in their responses to phosphorus limitation, heterotrophs displayed species-specific expression of nitrogen acquisition genes. These results suggest niche partitioning around nitrogen sources may structure the community when organisms directly compete for limited phosphate. Such niche complementarity around nitrogen sources may increase community diversity and productivity in phosphate-limited phototrophic communities.

## Introduction

Lack of mechanistic understanding of energy and element flow through microbial communities relegates them to black boxes in predictive models of ecosystem functioning (Nazaries et al., 2013; Graham et al., 2014; Rousk and Bengtson, 2014). Understanding the variables that control resource acquisition and partitioning in dynamic microbial communities is central to our ability to predict how biogeochemical cycles will respond to environmental change (Konopka et al., 2015). This is especially true for benthic microbial communities, in which energy and element cycling are affected by both members’ abundances and activities and physicochemical gradients on micron scales (Battin et al., 2003; Moran et al., 2014). In aquatic systems, phototrophic biofilms (“periphyton”) frequently serve as an ecosystem entry point for energy, carbon, and other macronutrients (Vadeboncoeur and Steinman, 2002; Kautza et al., 2016) and exert significant impacts on nutrient fluxes and carbon cycling at fluvial scales (Flipo et al., 2004; Battin et al., 2008). In these multi-species biofilms, consisting of photoautotrophs and associated heterotrophic consorts encased in a biogenic matrix of extracellular polymers (Roeselers et al., 2008), exchange of macronutrients between autotrophs and heterotrophs drives community succession (Wagner et al., 2015).

Though much research has been directed at understanding nutrient uptake by phototrophic biofilms at whole-community scales (Battin et al., 2016), little is known about how individual members acquire and exchange nutrients. This has resulted, in part, from an inability to resolve metabolic function at the level of individual species. However, recent advances in the reconstruction of individual genomes from metagenomes now permit the assignment of potential function at the species level (Pope et al., 2011; Hugerth et al., 2015; Palomo et al., 2016). We have applied these approaches to generate species-resolved metagenomes for two unicyanobacterial consortia (Nelson et al., 2016). These consortia are each composed of one distinct cyanobacterium and a nearly identical suite of ∼18 heterotrophic species from *Alphaproteobacteria*, *Gammaproteobacteria*, and *Bacteroidetes*, which form a benthic biofilm that undergoes a reproducible succession in the laboratory (Cole et al., 2014). Using these model systems, we predicted entry points for energy and elements into phototrophic biofilm communities by identifying each species’ functional potential for light energy capture and acquisition of the macronutrients carbon, nitrogen, phosphorus, and sulfur. Metatranscriptomic and metaproteomic analyses enabled attribution of energy and macronutrient acquisition processes at the level of individual species during succession. We observed common responses across the community to some nutrients (e.g., phosphorus) as well as highly individual strategies for others (e.g., nitrogen). These data allow a mechanistic understanding of community nutrient flow and suggest that niche complementarity or plasticity centered around nitrogen may minimize competition, maintaining diversity in phototrophic biofilms when organisms directly compete for limited phosphorus resources.

## Materials and Methods

### Consortia and Succession Experiments

Unicyanobacterial consortia UCC-A and UCC-O were isolated from a phototrophic microbial mat in Hot Lake, Washington (Lindemann et al., 2013) and were cultivated and sampled for succession experiments as previously described (Cole et al., 2014). Briefly, sequentially passaged enrichment cultures were inoculated at a 1:50 dilution into HLA medium (essentially, BG-11 medium supplemented with 400 mM MgSO_4_, 100 mM Na_2_SO_4_, and 25 mM KCl at pH 8.0) in T75 tissue culture flasks with vented caps (Costar, Corning, Inc., Corning, NY, United States) under 35 μE m^-2^ s^-1^ (General Electric PL/AQ, Fairfield, CT, United States). Cultures were incubated for 28 days and sterile deionized water was added weekly to maintain constant volume and salinity, with triplicate biological replicates (parallel T-75 flasks) harvested and split for proteomic and transcriptomic analysis at weekly intervals as previously described (on days 7, 14, 21, and 28 of cultivation; Cole et al., 2014). Briefly, biofilms were dislodged from the bottom of an entire T-75 flask using a cell scraper (Costar; Corning, Inc., Corning, NY, United States), placed into a 50 mL conical vial, and homogenized using sterile 3 mm glass beads and hand shaking, divided into separate samples for transcriptomics and proteomics, and centrifuged at 4°C and 5000 × *g* for 10 min prior to decanting and plunge freezing in liquid N_2_.

### Species-Resolved Genome Information

**FIGURE 1:**
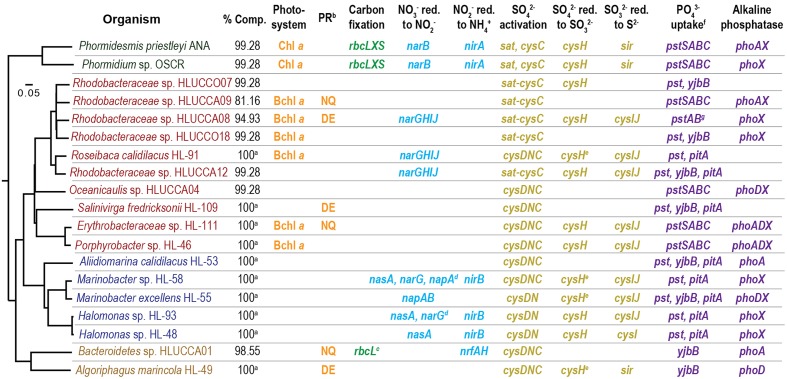
Macronutrient acquisition functions detected in phototrophic consortium member genomes. Isolates belonging to the *Cyanobacteria* are green, *Alphaproteobacteria* are red, *Gammaproteobacteria* are blue, and *Bacteroidetes* are yellow. ^a^Genomic information derived from sequenced isolates. ^b^For description of the types of proteorhodopsins, see Supplementary Notes 2. ^c^Similar to form IV RuBisCo, and thus unlikely to permit autotrophy (Supplementary Notes 3). ^d^These organisms contain multiple complete nitrate reductases; only one gene of each type of multi-subunit nitrate reductase is denoted to conserve space. ^e^Where organisms have multiple phosphate-acquisition systems, *pst* denotes the presence of *pstSABC*. ^*f*^The *pstAB* genes are contiguous to a contig edge in the *Rhodobacteraceae* sp. HLUCCA08 reconstruction; *pstSC* are assumed to be within the gap between contigs.

Metagenomes for UCC-A and UCC-O and genomes of isolates therefrom (*Algoriphagus marincola* HL-49, *Aliidiomarina calidilacus* HL-53 (Morton et al., in review), *Erythrobacteraceae* sp. HL-111, *Halomonas* sp. HL-48, *Halomonas* sp. HL-93, *Marinobacter excellens* HL-55, *Marinobacter* sp. HL-58, and *Porphyrobacter* sp. HL-46, *Roseibaca calidilacus* HL-91 (Maezato et al., in review), and *Salinivirga fredricksonii* HL-109 (Cole et al., in review) were generated by the DOE Joint Genome Institute, and species-resolved genome reconstructions were recently described (Nelson et al., 2016). These were evaluated for the presence of genes involved in light energy, carbon, nitrogen, phosphorus, or sulfur acquisition under experimental conditions. Genome completeness estimates from single-copy-gene analysis (Nelson et al., 2016) are presented in **Figure 1**; *Rhodobacteraceae* sp. Bin24 was excluded from further analysis due to insufficient completeness. A list of the accession numbers for genome reconstructions from metagenomes (European Nucleotide Archive^1^) and isolate genomes (GenBank^2^) are provided in **Supplementary Table S1** and are also available through IMGer^3^.

### Protein Function Predictions

Protein-coding gene models and functional predictions were initially assigned by the DOE-JGI microbial genome annotation pipeline (Markowitz et al., 2014) and manually curated by evaluating additional evidence collected by submitting these gene models to RAST (Aziz et al., 2008; Overbeek et al., 2014; Brettin et al., 2015) and BlastKoala (Kanehisa et al., 2016) as well as conducting local domain searches for TIGRfams (v. 14) and Pfams (v. 27) using HMMer v. 3.1 (Johnson et al., 2010). Identified genes involved in energy and macronutrient acquisition are provided in **Supplementary Table S2**. Note that, except for isolates where complete genomes are available, missing functions could reside in the gaps between contigs despite the near-completeness of genome reconstructions. Consequently, our predictions of organismal function are necessarily conservative, especially since some functions could require only a small number of genes that are frequently found together in operons. For details on genome annotation, please see the Supplementary Notes.

### RNA Extraction

RNA was extracted using Invitrogen TRIzol Reagent (cat. #15596018), followed by genomic DNA removal and cleanup using Qiagen RNase-Free DNase Set kit (cat. #79254) and Qiagen Mini RNeasy kit (cat. #74104). An Agilent 2100 Bioanalyzer was used to assess the integrity of the RNA samples; only RNA samples having RNA Integrity Number score of 8–10 were sequenced.

### RNA Sequencing

The Applied Biosystems SOLiD Total RNA-Seq kit (cat. #4445374) was used to generate the cDNA template library according to manufacturer’s instructions. The SOLiD EZ Bead system was used to perform emulsion clonal bead amplification to generate bead templates for sequencing on the 5500XL SOLiD platform. The 50-base read sequences produced by the 5500XL SOLiD sequencer were mapped in color space using SOLiD LifeScope software version 2.5 using the default parameters against an artificial chromosome, as previously described (Hess et al., 2013). LifeScope has been previously determined to be the optimal method for mapping sequence data obtained via the SOLiD 5500 system (Pranckevičiene et al., 2015), but it is optimized for use with eukaryotic genomes with multiple linear chromosomes. Consequently, we constructed an artificial chromosome as a reference by concatenating genomes of isolated UCC members and all contigs of remaining metagenome bins. These were separated by ten ambiguous nucleotides to prevent edge effects that might otherwise disturb mapping near contig boundaries. This reference contained species-resolved genome information for all consortium members. Transcription of each organism’s genes was individually normalized to reads per kilobase per million reads (RPKM), using the total reads mapped to all the organism’s genes as the normalization basis. It should be noted that though this normalization approach facilitates gene expression comparisons within a single species across time points, normalized gene expression cannot be compared across species. The transcriptomics data associated with this study has been deposited in the National Center for Biotechnology Information’s Gene Expression Omnibus under accession number GSE99220.

### Global Proteomics: Extraction, Digestion, and 2D-LC-MS/MS Analysis

UCC-O cell pellets (typically ∼100–500 mg, dry weight) were suspended in 100 mM NH_4_HCO_3_ buffer (pH 8.0) and then subjected to bead beating in a Bullet Blender homogenizer (Next Advance Inc., Averill Park, NY, United States) for 3 min with 0.1 mm zirconia/silica beads (BioSpec Products, Inc., Bartlesville, OK, United States). Proteins were separated into global, soluble, and insoluble fractions and processed into peptides for subsequent LC-MS/MS analysis using previously described methods (Callister et al., 2006). Briefly, proteins designated for global analysis were denatured and reduced under following conditions: 7M Urea, 5 mM DTT, 100 mM ammonium bicarbonate buffer at 60°C for 45 min. After denaturing, samples were diluted eightfold with 100 mM ammonium bicarbonate and a sufficient amount of calcium chloride was added to achieve 1 mM. Tryptic digestion was performed for 3 h at 37°C with 1:50 (w/w) trypsin-to-protein ratio. The digested sample was desalted and cleaned via solid phase extraction (SPE) C18 (Supelco, Bellefonte, PA, United States). Sample was concentrated in Speed-Vac (Thermo Savant, Holbrook, NY, United States) before performing a BCA Assay to determine final peptide concentration. A portion of the lysate for soluble/insoluble analysis was ultracentrifuged and the supernatant was treated as above, whereas the pellet was resuspended by sonication in denaturing buffer containing 1% CHAPS in 50 mM ammonium bicarbonate (pH 7.8) before enzymatic digestion. On line 2D-LS-MS/MS analysis of the peptides was achieved by using previously described methods (Smith et al., 2014). MS analysis was performed using a LTQ Orbitrap mass spectrometer (Thermo Scientific, San Jose, CA, United States) operated as described by Callister et al. (2006). The mass spectrometry proteomics data have been deposited to the ProteomeXchange Consortium via the PRIDE (Vizcaíno et al., 2016) partner repository with the dataset identifier PXD006440 and doi: 10.6019/PXD006440.

### MS/MS Data Analysis

MS/MS data were searched against protein sets for all organisms present in UCC-O (Nelson et al., 2016), a total of 60,759 proteins. Protein sets were augmented by common contaminant protein sequences (human keratin, bovine trypsin, and serum albumin precursor) to detect residual peptides derived from processing. MS/MS spectra were preprocessed using DeconMSn and DtaRefinery tools to deconvolute, deisotope, and remove systematic error in mass measurement accuracy (Mayampurath et al., 2008; Petyuk et al., 2010). The MS-GF+ search algorithm was used to match MS/MS spectra to peptide sequences (Kim et al., 2008), including partial tryptic cleavage peptides, dynamic modification of methionine oxidations, and maximum 10 ppm parent ion mass tolerance in the search. IDPicker 3.0 was used to filter peptide-spectrum matches to 2% FDR and apply parsimony filtering to derive a minimum protein list, with each protein supported by at least two distinct peptides (Ma et al., 2009; Tabb et al., 2010). For *Phormidium* sp. OSCR, proteomic coverage was sufficient to holistically describe gene expression; consequently, spectral counts of all peptides of a protein were summed and are presented as percentage of this species’ share of peptides observed at each time point.

### Fluorescence *In Situ* Hybridization (FISH)

Species-specific fluorescence probes were designed using the DECIPHER R package (Wright et al., 2014). Briefly, a list of the targeted 16S sequences and a FASTA file of potential off-target 16S sequences was generated from the UCC-O genome sequence (Nelson et al., 2016). Optimal specific probes were then generated with a length between 18 and 21 nucleotides, and a 46°C hybridization temperature in 0.9M NaCl and 20% formamide. This yielded probe GATACCCGAAAGCATCTCT for HL-49. Specificity of the probe was experimentally validated using mixtures of axenic cultures derived from the UCC-O biofilm. FISH of biofilms grown for 7 days was performed following the method described by Amann and Fuchs (2008). After FISH hybridization, samples were stained with Hoechst 33342 (Sigma, St. Louis, MO, United States) to stain DNA. Microscopic images were acquired on a Zeiss LSM 710 Scanning Confocal Laser Microscope (Carl Zeiss MicroImaging GmbH, Jena, Germany) for both fluorescence and differential interference contrast imaging. Z-stack confocal images were acquired using an EC Plan-Neofluar 10x/0.30 M27 objective. DNA fluorescence was excited at 405 nm visualized at 410–495 nm. FISH fluorescence was excited at 561 nm visualized at 566–680 nm. Phycocyanin/chlorophyll auto-fluorescence was excited at 633 nm and visualized at 647–721 nm. The images were further processed with Volocity (Perkin Elmer, Waltham, MA, United States).

## Results and Discussion

### Genome Predictions for Energy and Macronutrient Acquisition Potential of Species

We utilized our previously generated, species-resolved metagenome analysis (Nelson et al., 2016) to predict each consortium member’s genetic potential to harvest light energy and to directly acquire the required macronutrients carbon, nitrogen, phosphorus, and sulfur from the inorganic sources present in the culture medium (HLA; Cole et al., 2014). An overview of functions predicted within each member’s genome is shown in **Figure 1** and indicated by its type (e.g., Chl *a* vs. Bchl *a*-based phototrophy) or its keystone genes; the full list of genes important for these functions is detailed in **Supplementary Table S2**.

#### Energy

The consortia are routinely grown with light as the only energy source. Although oxygenic phototrophs are the main sources of energy capture by the consortia (Cole et al., 2014), several heterotrophic members encode bacteriochlorophyll *a* (Bchl *a*)-based photosystems. Such systems may operate under anoxic conditions, using reduced substrates (e.g., sulfide, fumarate) as electron donors for carbon fixation, or aerobically, driving cyclic transport of electrons to generate ATP. However, no Bchl *a*-containing photosystem has yet been shown to be capable of both anaerobic and aerobic phototrophy (Yurkov and Beatty, 1998; Rathgeber et al., 2012). Sequence analysis alone is incapable of differentiating anaerobic from aerobic photosystems, as both contain structurally and phylogenetically similar reaction centers and light-harvesting antenna complexes (Yurkov and Csotonyi, 2009). Multiple *Rhodobacteraceae* spp. (*Roseibaca calidilacus* HL-91 and HLUCCA08, HLUCCA09, and HLUCCO18) and *Erythrobacteraceae* spp. (HL-111 and *Porphyrobacter* sp. HL-46) in the consortia possess all the genes required for Bchl *a* synthesis and photosystem assembly. Under continuously illuminated oxic growth conditions, we postulate that only organisms with aerobic photosystems (aerobic anoxygenic phototrophs, or AAPs) are likely to supplement cyanobacterial light energy acquisition (Cole et al., 2014). However, because AAP photosystems do not generate the reductant required to fix inorganic carbon (Yurkov and Beatty, 1998), they are constrained to photoheterotrophy or chemoheterotrophy.

One additional means by which light energy could be captured by the consortia is through bacterial proteorhodopsins (PRs), which are transmembrane proteins that use photons to drive proton or other ion gradients that generate ATP and energize membrane transporters (McCarren and DeLong, 2007). PR-like proteins are encoded by heterotrophic members of *Bacteroidetes* (HLUCCA01 and *A. marincola* HL-49) and *Alphaproteobacteria* (*Rhodobacteraceae* spp. HLUCCA08 and HLUCCA09, *Salinivirga fredricksonii* HL-109, and *Erythrobacteraceae* sp. HL-111). In *Bacteroidetes* sp. HLUCCA01, *Rhodobacteraceae* sp. HLUCCA09, and *Erythrobacteraceae* sp. HL-111, the putative rhodopsin contains the RYXN(X_10_)Q transport motif characteristic of the NQ family of rhodopsins rather than the RYXD(X_10_)E proton-transport motif; NQ rhodopsins are common in hypersaline environments (Kwon et al., 2013) and have recently been shown to transport sodium ions (Balashov et al., 2014). This suggests that rhodopsins may perform other functions besides maintaining proton-motive force for ATP generation in these organisms, such as regulating osmotic pressure or driving efflux pumps via cation antiport (Fuhrman et al., 2008). Consequently, it is possible that these PR-containing heterotrophs could harvest light energy although recent theoretical work suggests the net energetic advantage of PR-containing bacteria is significantly smaller than for AAPs (Kirchman and Hanson, 2013).

#### Carbon

Genomic evidence supports our previous hypothesis that the cyanobacteria were the sole autotrophs within the consortia (Cole et al., 2014). Both cyanobacteria contained the genes (*rbcL, rbcS*) required to construct the canonical, hexadecameric ribulose-1,5-bisphosphate carboxylase/oxygenase (RuBisCo). RuBisCo catalyzes the addition of carbon dioxide to ribulose-1,5-bisphosphate and is required for the Calvin-Benson-Bassham reductive pentose phosphate cycle of carbon fixation (Tabita et al., 2007; Erb et al., 2012). Although *Bacteroidetes* sp. HLUCCA01 also contains an *rbcL* homolog, its catalytic motif is similar to the form IV RbcLs of *Rhodopseudomonas palustris* (gi: 77687805) and *Rhodospirillum rubrum* (gi: 48764419) as it is ∼100 residues shorter than form I-III RbcLs and His replaces the canonical Glu^204^ residue in the catalytic motif (Carré-Mlouka et al., 2006). Form IV RuBisCos, also termed RuBisCo-like proteins, have been found to lack the ability to fix carbon dioxide but instead catalyze an enolization reaction important for the salvage of methionine from methylthioadenosine (Tabita et al., 2007; Erb et al., 2012). Consequently, this gene is unlikely to enable autotrophy in *Bacteroidetes* sp. Bin01. HLUCCA01.

Although it is likely that the vast majority of the inorganic carbon entering the consortia comes through the cyanobacterial primary producers, a modest amount of carbon could enter the community through the anapleurotic reactions of heterotrophs and, especially, of photoheterotrophs. For example, *Roseobacter denitrificans* has been shown to acquire 10–15% of its protein carbon through the activities of pyruvate carboxylase and/or phosphoenolpyruvate (PEP) carboxylase (Tang et al., 2009). All members of the consortia encoded at least one mechanism to convert PEP or pyruvate to oxaloacetate through the incorporation of carbon dioxide or bicarbonate (i.e., either pyruvate or PEP carboxylase) except *A. calidilacus* HL-53.

#### Nitrogen

Because the consortia are routinely cultivated with abundant nitrate (17.6 mM), the primary organisms through which nitrogen enters the consortia will be those capable of reducing nitrate to nitrite to bioavailable ammonium (Cole et al., 2014). Although both *Phormidium* sp. OSCR and *Phormidesmis priestleyi* ANA also contain *nif* genes encoding the MoFe nitrogenases that catalyze the reduction of N_2_ to ammonium, the expression of nitrogenases is typically strongly repressed in cyanobacteria when nitrate or ammonium are available (e.g., Bottomley et al., 1979; Mackerras and Smith, 1986), as under these growth conditions. Both cyanobacterial genomes possess ferredoxin-dependent, cytoplasmic nitrate and nitrite reductases, allowing them to serve as primary producers with respect to nitrogen acquisition. However, nitrate assimilation is not uniformly distributed among bacteria. Thus, though we originally hypothesized that our enrichment conditions would select for nitrate-assimilating heterotrophs, genomic analysis suggested that the majority of heterotrophic species lacked the ability to reduce nitrate, nitrite, or both. Only the gammaproteobacterial genomes, except for *A. calidilacus* HL-53 and *M. excellens* HL-55, contained cytoplasmic NO_3_^-^ nitrate (*nasA*) and nitrite reductases (*nirB*) required for assimilatory reduction. In addition to assimilatory reductases, some gammaproteobacterial members also contained dissimilatory nitrate reductase genes, which have been shown to substitute for *nasA* in some species (Moreno-Vivian et al., 1999); *Halomonas* sp. HL-93 contained the membrane-bound respiratory reductase *narGHIJI*, *M. excellens* HL-55 possesses the periplasmic *napAB* system, and *Marinobacter* sp. HL-58 encoded both types. Alphaproteobacterial members *Rhodobacteraceae* spp. HL-91, HLUCCA08, and HLUCCA12 also encoded *narGHIJ* for dissimilatory reduction of nitrate to nitrite but no predicted nitrite reductases. Conversely, *Bacteroidetes* sp. HLUCCA01 contained the dissimilatory, ammonifying nitrite reductase *nrfAH* but appeared to lack a nitrate reductase. All members of the consortia contained Amt-like ammonium transporters and glutamine synthetases required to incorporate ammonium.

#### Sulfur

Although all consortium members were predicted to transport and activate sulfate, only a subset appeared to be able to reduce sulfate to sulfide for the biosynthesis of cysteine and methionine, thereby serving as entry points for sulfur into the community. All members were capable of activating sulfate to 3′-phosphoadenylyl-sulfate (PAPS), which generates a high-energy phosphoric-sulfuric acid anhydride bond and allows transfer or reduction of the sulfurylyl group (Leyh et al., 1988). *Roseibaca calidilacus* HL-91, *Marinobacter* sp. HL-58, *Marinobacter excellens* HL-55, and *Algoriphagus marincola* HL-49 encode thioredoxin-dependent adenosine 5′-phosphosulfate (APS) reductases (TIGR2055 family); these enzymes are capable of reducing both APS and PAPS to sulfite. However, *Bacteroidetes* sp. HLUCCA01, *A. calidilacus* HL-53, *Oceanicaulis* sp. HLUCCA04, *S. fredricksonii* HL-109, and *Rhodobacteraceae* spp. HLUCCA09 and HLUCCO18 all were predicted to lack both PAPS and sulfite reductases, which are required to produce sulfide for cysteine biosynthesis. Consequently, these members are predicted to depend upon sulfate assimilators for acquisition of bioavailable sulfur under routine culture conditions. Since these organisms are derived from a phototrophic mat, regions of which are transiently or permanently sulfidic (Lindemann et al., 2013), it is possible that organisms without the ability to reduce PAPS are able to directly acquire sulfide from the native environment for cysteine and methionine biosynthesis.

#### Phosphorus

Due to the extremely high concentrations of Mg^2+^ in Hot Lake and in culture medium formulated to cultivate mat-associated microorganisms, inorganic phosphorus is sparingly soluble (Lindemann et al., 2013; Cole et al., 2014; Zachara et al., 2016). The types of phosphate transporters (Willsky and Malamy, 1980; Rao and Torriani, 1990) possessed by members of the consortia presented a genomic signature of competition for phosphate; although high-affinity, ATP-utilizing PstSABC-like transporters were found in all members except both of the *Bacteroidetes*, low-affinity, high-rate PiT-like proton:phosphate symporters are encoded only in seven of the nineteen examined genomes. Both members from *Bacteroidetes* relied upon phosphate:sodium symporters of the PNaS family (termed *yjbB*; Lebens et al., 2002) for phosphate uptake, which were also present in six other genomes. The insolubility of magnesium and calcium phosphates suggests that the majority of community phosphorus exchange may occur via salvage of phosphorus-containing organic compounds or phosphatase-mediated removal of orthophosphate from inorganic phases. All but four alphaproteobacterial member species (*S. fredricksonii* HL-109, *Roseibaca calidilacus* HL-91, and *Rhodobacteraceae* spp. HLUCC07 and HLUCC12) encoded at least one alkaline phosphatase.

### Species-Resolved Macronutrient Acquisition during Succession

**FIGURE 2:**
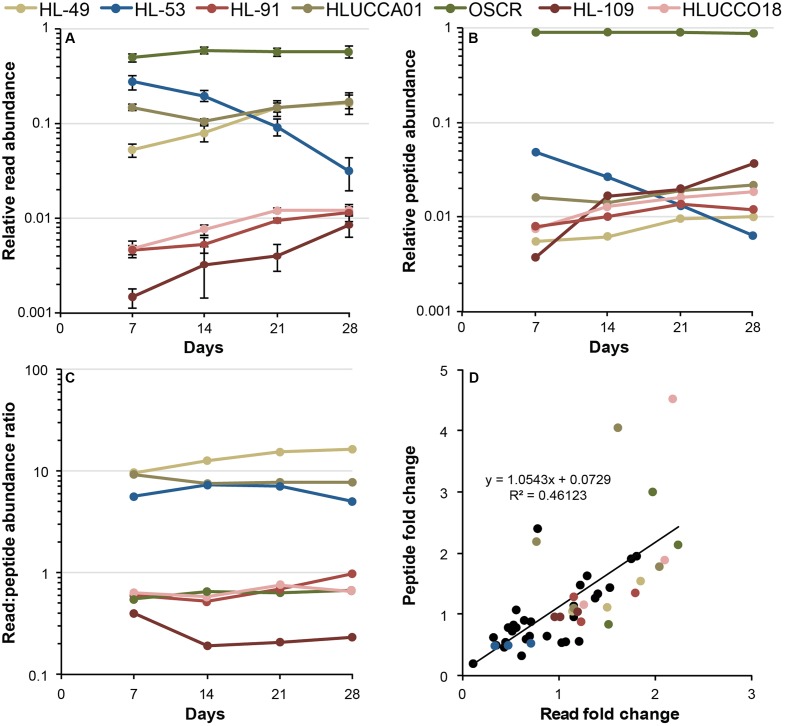
Metatranscriptomic and metaproteomic measures of member activity dynamics. **(A)** Metatranscriptomic measurement of member activity during succession, represented as the fraction of the total reads per sample attributed to each organism. Values are plotted on a log_10_ scale, error bars denote the 95% confidence interval of the mean. **(B)** Metaproteomic evaluation of member activity during succession, represented as the share of peptide spectral counts attributed to each of the members. Values are plotted on a log_10_ scale. Error bars are omitted for clarity, but variance data are presented in **Supplementary Table S3**. **(C)** Ratio of member relative activity as assessed by metatranscriptomics to that observed by metaproteomics during succession. Flat lines (*m* = 0) indicate a monotonic relationship between metatranscriptomic and metaproteomic estimations. **(D)** Organism-centric changes in metatranscriptomic and metaproteomic estimates of relative activity across all members between sequential time points. The *y*-axis indicates the between-time point fold change in the relative abundance of an organism’s peptide spectral counts, and the *x*-axis indicates the fold change in an organism’s RNA read abundance. Colored dots represent the seven most dominant species according to the key, and black dots indicate fold changes for less-abundant species.

Species-resolved genome information enabled reconstruction of the dynamics of member abundance and resource acquisition in UCC-O over a 28-day succession period (Cole et al., 2014) via metatranscriptomic and metaproteomic analysis (**Figure 2**). *Phormidium* sp. OSCR dominated both metatranscriptomic and metaproteomic analyses, averaging ∼50–60% of total mRNA reads (**Figure 2A**) and ∼90% of total peptide spectral counts (**Figure 2B**). This large share of cyanobacterial peptides allowed us to comprehensively evaluate the activity of *Phormidium* sp. OSCR via proteomics. Peptide spectral counts were insufficient to do this for any heterotroph. In general, the low RNA and protein abundances of some heterotrophic organisms greatly obscured their activity. Only six of the 17 heterotrophs were sufficiently represented throughout the succession cycle to comprehensively describe patterns in their gene transcription. The remaining eleven either could only be examined at a subset of time points or with respect to certain highly expressed functions, some of which were also represented in the metaproteome. We focus here on the six most abundant heterotrophs for which sufficient transcriptomics data exist to describe expression patterns over time (expression data for the referenced genes of all organisms are provided in **Supplementary Table S4**).

**FIGURE 3:**
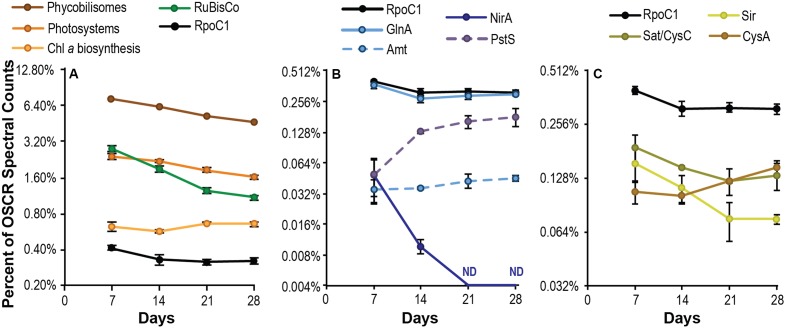
Cyanobacterial energy capture and macronutrient acquisition during succession. Expression of *Phormidium* sp. OSCR proteins involved in light energy capture and carbon fixation **(A)**, nitrogen and phosphorus acquisition **(B)** and sulfur acquisition **(C)**, expressed as a percentage of total spectral counts per sample attributed to this species. The expression of the large subunit of the cyanobacterial beta’ subunit of RNA polymerase, RpoC1, is provided for reference in all panels. Data are plotted on a log_2_ scale; error bars represent the 95% confidence interval of the mean. For energy and carbon capture systems **(A)**, data represent a sum of all the subsystem-specific cyanobacterial gene products notated in **Supplementary Table S2**.

**FIGURE 4:**
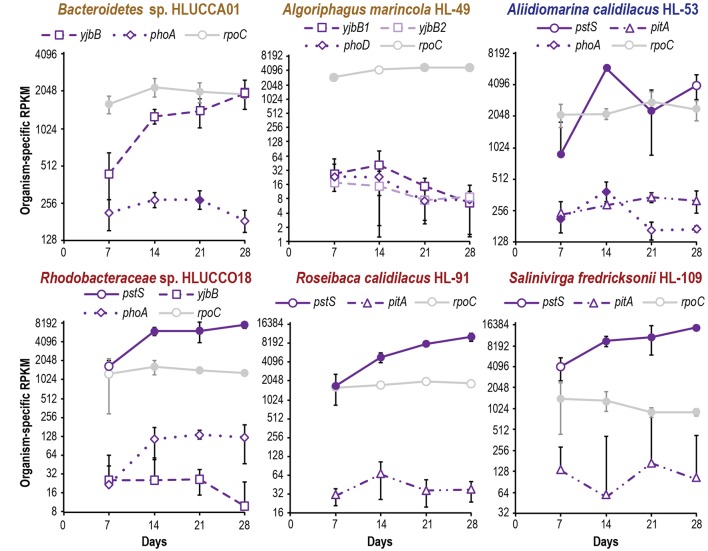
Metatranscriptomic evaluation of phosphate acquisition gene expression in major heterotrophic species during succession. Expression of the substrate-binding subunit *pstS* is plotted as an indicative gene for *pstSABC*, as other transporters and phosphates are each encoded by a single gene. In organisms where they are present, the *pst* genes are operonic and expression of *pstABC* displayed similar trends as *pstS* (**Supplementary Table S4**). Organism-specific RPKM is plotted on a log_2_ scale; error bars indicate 95% confidence interval of the mean (where the 95% confidence interval is inclusive of zero or below, negative error bars are not shown). Filled symbols indicate that two or more peptides were observed for a gene’s product at a given time point; open symbols denote no or inadequate proteomic evidence. Expression of *rpoC* is included as a reference.

**FIGURE 5:**
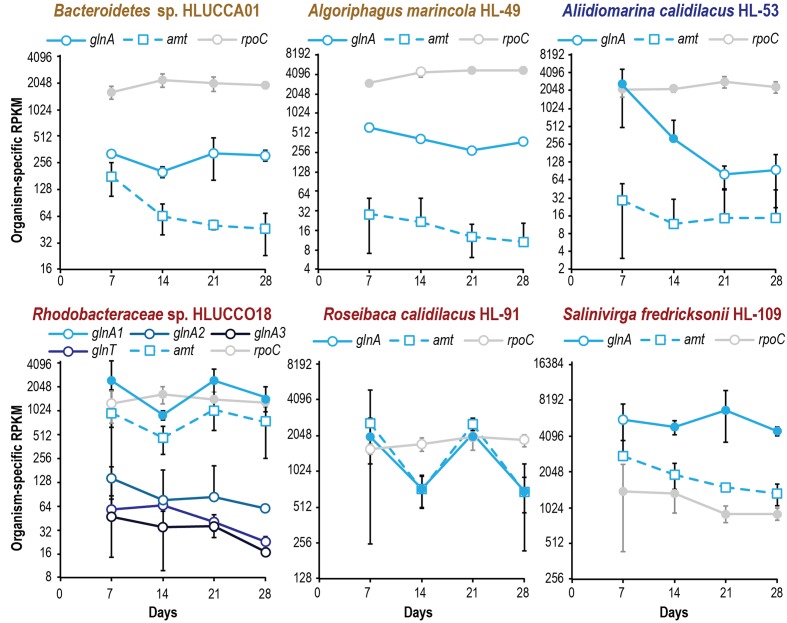
Metatranscriptomic evaluation of ammonium incorporation gene expression in major heterotrophic species reveals species-specific patterns of nitrogen limitation during succession. Organism-specific RPKM is plotted on a log_2_ scale; error bars indicate 95% confidence interval of the mean (where the 95% confidence interval is inclusive of zero or below, negative error bars are not shown). Filled symbols indicate that two or more peptides were observed for a gene’s product at a given time point; open symbols denote no or inadequate proteomic evidence. Expression of *rpoC* is included as a reference.

Metatranscriptomics and metaproteomics results were in agreement on the trends in member abundance between time points, but displayed large differences in their estimation of the relative gene expression activity (i.e., the fraction of the community’s total mRNA or protein attributable to a given species) across the consortium members (**Figure 2C**; also see **Supplementary Table S3**). In general, the trends in relative gene expression activity (**Figures 2A,B**) matched our previous examination of relative genome abundance through succession cycles, notably the replacement of *A. calidilacus* HL-53 and other *Gammaproteobacteria* with members from *Alphaproteobacteria* and *Bacteroidetes* (Cole et al., 2014). However, estimates of gene expression activity from metatranscriptomics and metaproteomics differed substantially for some members. The ratio of relative gene expression activity to corresponding proteins from paired samples were higher than unity (i.e., larger metatranscriptomic estimates of abundance) for *A. marincola* HL-49, *Bacteroidetes* sp. HLUCCA01, and *A. calidilacus* HL-53 and lower (i.e., larger metaproteomic estimates of abundance) for *Phormidium* sp. OSCR, *R. calidilacus* HL-91, *Rhodobacteraceae* sp. HLUCCO18, and *S. fredricksonii* HL-109. The transcript (read)/protein (peptide) relative abundance ratios ranged nearly 100-fold across the most abundant organisms (a maximum of ∼16.6 for HL-49 and minimum of ∼0.2 for HL-109) and were relatively stable throughout the succession (**Figure 2C**). Overall, the inter-sample transcript fold change (e.g., between days 7 and 14, 14 and 21, and 21 and 28 of each of the 18 organisms correlated relatively well to inter-sample protein fold change (**Figure 2D**), exhibiting a slope near unity (with an *R*^2^ ∼ 0.46). Where discrepancies existed, changes in an organism’s share of the metaproteome tended to be larger than corresponding changes in the metatranscriptome (per-species transcript and protein abundance data are presented in **Supplementary Table S3**). To eliminate the influence of changes in member relative abundance (**Figures 2A,B**) that might otherwise obscure individual species’ gene expression patterns, mRNA reads and peptide spectral counts for all genes were normalized to the total number reads/peptides attributed to that species on a per-sample basis (see Materials and Methods). The relatively constant expression level of the highly conserved housekeeping gene *rpoC* over time across multiple species supports our use of this normalization approach (**Figures 3**–**5** and **Supplementary Table S4**).

Metaproteomic analysis revealed large shifts in energy and macronutrient acquisition by the cyanobacterium *Phormidium* sp. OSCR during succession. As a share of its total proteome, abundance of both light-harvesting complexes (photosystems I and II) and antenna complexes (phycobiliproteins) declined steadily over time (**Figure 3A**), ending on day 28 at 66.5 and 63.6% of their respective day 7 totals and suggesting reduced per-cell light-energy capture. In contrast, production of chlorophyll *a* biosynthesis proteins remained steady. RuBisCo abundance declined over the 28-day succession, to 38.4% of the day 7 relative abundance. This indicates a substantially decreased per-cell fixation of inorganic carbon by the end of the experiment. Similarly, initially high expression of bacteriochlorophyll *a* synthesis and photosystem genes by photoheterotrophic species (*R. calidilacus* HL-91, *Porphyrobacter* sp. HL-46, and *Erythrobacter* sp. Bin15) declined ∼four-fold during succession, suggesting reduced per-cell energy capture by these species as well (**Supplementary Table S4**). Nitrate assimilation by *Phormidium* sp. OSCR, as measured by NirA abundance, decreased concurrently with RuBisCo and was below detection by day 21. In contrast to the substantial reduction in nitrate assimilation proteins, ammonium uptake via Amt and incorporation via GlnA were relatively stable during succession, varying less than 1.5-fold (**Figure 3B**). We also observed substantial declines in sulfate activation (Sat/CysC, reduced 31.0%) and sulfite reduction (Sir, reduced 51.3%) between days 7 and 28 (**Figure 3C**), though Cys synthase (CysA) was stable or slightly increased. A large increase in phosphate transporter abundance, as assessed by expression of the substrate-binding subunit PstS, suggested phosphate scarcity.

Metatranscriptome analysis indicated that all but one of the dominant heterotrophic members also showed signs of increasing phosphate scarcity during succession. Although it is important to note that differences in translation efficiency (Taylor et al., 2013), post-transcriptional regulation, and protein and transcript turnover rates mean that mRNA abundance is an imperfect predictor of protein abundance (Waldbauer et al., 2012). However, transcript abundance reflects the intracellular and environmental signals to which an organism is responding. Transcripts for high-affinity *pst* phosphate transporters displayed approximately four-fold increases during succession in *A. calidilacus* HL-53, *Rhodobacteraceae* sp. HLUCCO18, *R. calidilacus* HL-91, and *S. fredricksonii* HL-109, and we observed similar increases in *yjbB* expression in *Bacteroidetes* sp. HLUCCA01 (**Figure 4**). PstS peptides were commonly observed, especially at later time points, and were among the few peptides observed from moderate-abundance members such as *M. excellens* HL-55, *Oceanicaulis* sp. HLUCCA04, and *Rhodobacteraceae* sp. HLUCCA09 (**Supplementary Table S4**), suggesting high expression within those organisms. Alkaline phosphatase genes (*phoA*, *phoD*) were also substantially upregulated in *A. calidilacus* HL-53 and *Rhodobacteraceae* sp. HLUCCO18 (**Figure 4** and **Supplementary Table S4**), though *phoA* expression in *A. calidilacus* declined after day 14, perhaps due to a reduced phosphate requirement late in the growth period. Peptides from alkaline phosphatases were observed for *Bacteroidetes* sp. HLUCCA01 and *A. calidilacus* HL-53 (PhoA) and for low-abundance member *Erythrobacteraceae* sp. HL-111 (PhoD). In organisms expressing a Pst transporter, expression of YjbB-family symporters and low-affinity PitA transporters was much lower. These data suggested that, like the cyanobacterium, most heterotrophic community members were responding to phosphate limitation by day 14. This is interesting in that UCC-O’s biomass continued to increase nearly linearly throughout the 28-day experimental period and at day 14 is only approximately half its final value (Cole et al., 2014), suggesting these organisms respond transcriptionally to declining phosphate long before it becomes limiting for growth. Such a response may confer fitness in Hot Lake, where extreme competition for phosphate is likely, due to increases in magnesium concentrations throughout the seasonal cycle (Lindemann et al., 2013). In contrast, *A. marincola* HL-49 displayed stably low expression of both of its *yjbB* genes as well as *phoD*, suggesting this organism did not experience phosphorus limitation. In contrast to increasing phosphate transporter expression, heterotrophic expression of genes for sulfate activation, reduction of PAPS and sulfite, and cysteine synthesis was generally stable over the same period for all members (**Supplementary Table S4**).

Though unified (excepting *A. marincola* HL-49) in their expression patterns of phosphate and sulfate acquisition genes, the dominant heterotrophic members of UCC-O exhibited very divergent responses in expression of nitrogen acquisition genes. The abundance of gammaproteobacteria capable of assimilating nitrate were very low throughout the growth period and provided no conclusive evidence of *nasA* or *nirB* expression; consequently, the majority of community nitrate assimilation was assumed to be cyanobacterial. We therefore used expression of genes involved in ammonium uptake (*amt*-family transporters) and incorporation (glutamine synthetases *glnA* and *glnT*) as markers of heterotrophic nitrogen starvation, as these genes are known to be induced under nitrogen limitation in model organisms (Hervás et al., 2008; Zimmer et al., 2000). In *Rhodobacteraceae* spp. HLUCCO18 and *R. calidilacus* HL-91, *glnA* and *amt* were tightly co-expressed (**Figure 5**) but were decoupled in *Bacteroidetes* sp. HLUCCA01, *A. calidilacus* HL-53, and *S. fredricksonii* HL-109. Both *Bacteroidetes* sp. HLUCCA01 and *S. fredricksonii* HL-109 exhibited high and stable expression of *glnA* and high and declining transcription of *amt*, respectively, suggesting nitrogen limitation for these species was greatest early in succession and declined thereafter. Coexpression of *amt* and the major *glnA* genes in the *Rhodobacteraceae* spp. alternated between highs on days 7 and 21 and lows on days 14 and 28, suggestive of alternating nitrogen-deplete and -replete conditions for these two species. Alternate glutamine synthetase genes (*glnA2, glnA3*, and *glnT*) in *Rhodobacteraceae* sp. HLUCCO18 were poorly expressed and did not show significant variation over time. Similarly, ammonium transporters were poorly expressed in *A. marincola* HL-49 and *A. calidilacus* HL-53 despite consistently high expression of *glnA* in HL-49 and initially high expression in HL-53. Expression of the *A. calidilacus glnA* declined precipitously (∼25-fold) by day 28, suggesting dramatically reduced ammonium incorporation late in the succession period. These data suggested that *A. marincola* and *A. calidilacus* may acquire their nitrogen from nitrogenous organic compounds provided by other members rather than direct incorporation of deamination-derived ammonium, and that *A. calidilacus* may switch its major nitrogen source during succession. Taken together, these data suggest that, in contrast to response to phosphate limitation observed at the whole community level, nitrogen limitation is highly specific to individual species and impacts species differentially during succession.

### Linking Expression Patterns to Energy and Nutrient Cycling during Succession

Although community-level functional potential and gene expression patterns in aquatic, phototrophic biofilms have been examined previously (Klatt et al., 2013; Leary et al., 2014; Graham et al., 2015; Sanli et al., 2015), combining species-resolved metagenomics with metatranscriptomics and metaproteomics provides a new approach for predicting the dynamics of energy and nutrient cycling at the level of individual species within a community context. This approach predicts the routes by which energy and elements enter communities, which is key to understanding how their interactions influence community dynamics and biogeochemical cycles. In this work, we used a tractable, phototrophic consortium, for which complete, species-resolved genomic information is available, to predict which species could serve as net “importers” of community energy and elemental resources and to examine species-specific energy and nutrient responses over biofilm succession. As the UCC biofilms are closed systems except for light energy input and gas exchange, each replicate can be treated as an individual microecosystem with respect to nutrient cycling (Gorden et al., 1969). Despite sequential passage in medium containing only inorganic macronutrients, we found a surprising lack of genomic potential in the dominant community members to assimilate these resources. This likely reflects extensive metabolic interdependency among species, especially heterotroph dependence upon the cyanobacterium for acquisition of all macronutrients (excepting phosphorus).

We evaluated individual-member functional responses during succession using metatranscriptomic and metaproteomic analyses of identical samples. Although the two approaches agreed well on the direction and magnitude of within-organism changes in gene expression activity, they disagreed sharply in their estimation of relative activity across organisms. Interestingly, these patterns displayed taxon-specificity, with *Bacteroidetes* members exhibiting substantially greater relative abundances (∼10-fold) in the metatranscriptome than in the metaproteome. Transcript: protein abundance ratios were relatively stable over time, though member relative abundances in each measurement varied up to ∼10-fold, suggesting that these ratios are properties of the organisms and not solely the result of member abundances or growth rates (e.g., the transcript:protein ratio of *A. calidilacus* HL-53 remains stable despite a ∼10-fold reduction in relative abundance in each; **Supplementary Table S2**). Variation in this ratio potentially stems from differences in cell size and/or biomass, species-level variability in extraction efficiency for RNA versus protein, or organismal regulatory strategies; however, nearly 100-fold differences between transcript and protein abundances for some species suggests that extreme caution be exercised when using either approach to evaluate which microbial populations within a community are “active” (Siggins et al., 2012; Kolmeder and de Vos, 2014; Satinsky et al., 2015; Thureborn et al., 2016).

During succession, autotroph energy, carbon, nitrogen, and sulfur uptake decrease substantially, a phenomenon that has been long-observed in other phototrophic biofilms (Cooke, 1967). Phosphorus limitation in UCC-O by day 14, displayed by increased expression of phosphate acquisition genes across all members except *A. marincola* HL-49, likely caused a decline in the cyanobacterial growth rate and, consequently, the abundance of cyanobacterial inorganic carbon, nitrogen, and sulfur acquisition proteins. Despite this decline in acquisition of new, oxidized nitrogen (nitrate) and sulfur (sulfate) resources by *Phormidium* sp. OSCR late in succession, abundances of glutamine synthetase and cysteine synthase suggested stable incorporation of reduced nitrogen and sulfur into amino acids over the entire period. This suggests a shift late in succession from *de novo* synthesis toward cyanobacterial recycling of community reduced nitrogen and sulfur stores into amino acids. Notably, declines in cyanobacterial sulfate reduction were less extensive than for nitrate, suggesting the possibility of a loss process for bioavailable sulfur (i.e., biological or chemical sulfur oxidation) not present for nitrogen. Though transitions from open to closed biogeochemical cycles and reductions in energy capture as communities mature have been long-predicted (Odum, 1969), our data suggest that these transitions are mediated by divergent gene expression responses at the level of individual species. In this study, phosphate limitation appeared to usher in the transition from early successional phases with relatively high cellular growth rates and correspondingly high acquisition of oxidized resources (e.g., nitrate, sulfate) to more mature, slower growth phases where recycling of reduced forms appears to dominate. Though the mechanism for phosphate limitation in Hot Lake and its derived cultures (insolubility due to high Mg^2+^) is unusual, it should be noted that phosphorus scarcity is common in periphyton biofilms (Rejmánková and Komárková, 2000; McCormick et al., 2001; Borovec et al., 2010; Hagerthey et al., 2011).

**FIGURE 6:**
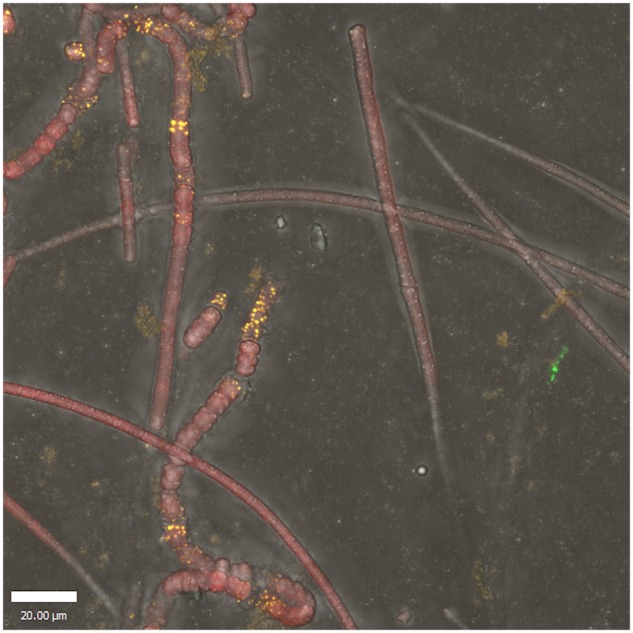
Localization of *A. marincola* HL-49 within UCC-O biofilms via fluorescence *in situ* hybridization. Image represents a maximum-intensity Z-projection of a stack of confocal images, scale bar denotes 20 mm. Fluorescence intensity from FISH probes targeted against *A. marincola* HL-49 16S ribosomal RNA is depicted in yellow. Cyanobacterial chlorophyll *a* and phycocyanin auto-fluorescence intensity appear in red within filaments of *Phormidium* sp. OSCR. Fluorescence images are overlaid upon a differential interference contrast image of consortium biomass to display cell boundaries.

The gene expression patterns of heterotrophic and photoheterotrophic consortium members mirrored those of the cyanobacterium with respect to energy, sulfur, and phosphorus acquisition, but diverged with respect to nitrogen acquisition. Species-specific expression of macronutrient acquisition genes was largely consistent with prior taxonomy-based predictions of member functional roles (Cole et al., 2014). It is noteworthy that the only organism that does not show elevated transcription of phosphate-acquisition genes during succession is *A. marincola* HL-49, which displays high *glnA* but very low *amt* expression. This suggests a detritivorous role in which it acquires its phosphorus from detrital nucleic acids. This suggestion is somewhat supported by the preferential localization of *A. marincola* HL-49 in UCC biofilms to cyanobacterial cells that appear to have compromised membrane integrity, lacking significant photopigment fluorescence and phase contrast (**Figure 6**). If true, this may make *A. marincola* HL-49 a potential keystone species for liberation of otherwise-inaccessible phosphorus resources. Nucleic acid turnover may therefore be an ecological role important for maximizing sustainable biomass in periphyton biofilms through increases in the velocity of phosphorus cycling.

Similarly to *A. marincola* HL-49, the hypothesized protein degrader *A. calidilacus* HL-53 (Hou et al., 2004) also exhibits low *amt* but initially high *glnA* expression, suggesting consumption of nitrogen-containing molecules (e.g., amino acids) as carbon and energy sources. As both *A. marincola* (Yoon et al., 2004) and *A. calidilacus* are likely to express extracellular proteases, either of these organisms may facilitate nitrogen availability to other members via deamination. A combination of declining population sizes and ammonium production of *A. calidilacus* HL-53 could also contribute to the day 21 nitrogen limitation of the *Rhodobacteraceae* spp. HLUCCO18 and HL-91. We hypothesize that differences in expression of ammonium incorporation genes across community members reflect species-specific consumption of different nitrogen sources that fluctuate in availability.

The predicted inability of many heterotrophs in the consortia to access oxidized forms of macronutrients, therefore relying upon metabolic exchange to supply these elements, concurs with our recent demonstration of a similar lack of acquisition systems in genomes reconstructed from Hot Lake-derived metagenomes (Mobberley et al., 2017). Of the 34 genome reconstructions reported in this study, 18 appeared to lack the ability to generate ammonium from more oxidized nitrogen sources and 23 lacked genes required for sulfate assimilation. That these genes are missing even within complete genomes from UCC heterotrophs (Nelson et al., 2016) further suggests that these functions are not lacking in mat metagenome-derived genome reconstructions solely due to incomplete genome information. Organisms lacking these functions were substantially more common at depth in the mat compared with overlying strata in closer communication with the water column (Mobberley et al., 2017). This is expected in that reduced forms of macronutrients (i.e., nitrogen, sulfur) that otherwise might be oxidized by other microbes as energy sources should be more stable in regions of the mat that are suboxic for at least some period. However, the data presented here suggest that stable metabolic interactions, in which heterotrophs are completely dependent upon other organisms for reduced macronutrients, occur reproducibly in ∼100 μm-thick phototrophic biofilms exposed to continuous light. We therefore submit that the ecological importance of such obligate interactions in phototrophic biofilms is not necessarily reflected by the fraction of reconstructed genomes predicted to lack acquisition systems. Furthermore, this study suggests that organismal specialization around different nitrogenous compounds may be a mechanism supporting maintenance of diversity as magnesium levels increase in Hot Lake and reduce phosphorous bioavailability.

Taken together, our results suggest the heterotrophs in UCC-O are generally phosphorus-replete but nitrogen-limited early in succession, after which phosphorus becomes limiting. They also suggest that the availability of distinct nitrogen sources might partition and structure diverse phototrophic microbial communities when phosphate, for which many organisms must directly compete, is sparingly available. In phosphorus-limited periphyton biofilms, niche partitioning around nitrogen sources may therefore help maintain the high diversity of these communities (Larson et al., 2016). As genome reconstruction from metagenome data becomes more accurate and omics measurements more sensitive and quantitative, it will enable similar studies in multiple phototrophic biofilm systems in the field to determine whether niche partitioning around nitrogen sources is a common community response to phosphate limitation in natural systems.

## Author Contributions

SL, JF, and MR designed the experiment, SL, JM, WN, and MR generated genome predictions, SL and JC performed succession experiments, LM, RT, and HW performed metatranscriptomic analyses, EH and ML performed metaproteomic analyses, SL, JM, and MR analyzed genome-resolved expression data, and all authors contributed to the manuscript.

## Conflict of Interest Statement

The authors declare that the research was conducted in the absence of any commercial or financial relationships that could be construed as a potential conflict of interest.

## Footnotes

1 www.ebi.ac.uk/ena

2 https://www.ncbi.nlm.nih.gov/genbank/

3 https://img.jgi.doe.gov/cgi-bin/mer/main.cgi

## Supplementary Material

TABLE S1 Accession numbers for unicyanobacterial consortium isolate genomes and genome reconstructions.

TABLE S2 Member genes predicted to be involved in energy or macronutrient acquisition.

TABLE S3 Per-sample RNA read and peptide abundances attributed to each consortium member genome.

TABLE S4 Expression of energy and macronutrient acquisition genes by each consortium member during succession.
