# Supplementary material for: Predicting Species-Resolved Macronutrient Acquisition during Succession in a Model Phototrophic Biofilm Using an Integrated ‘Omics Approach
Source: Front Microbiol. 2017 Jun 13;8:1020. doi: 10.3389/fmicb.2017.01020 (PMC5468372; doi:10.3389/fmicb.2017.01020)
Supplement: Supplementary file 5 [file Data_Sheet_1.docx]

***Supplementary Notes on Genome Annotation***

*Energy*

1. Sequence analysis alone is incapable of differentiating anaerobic from aerobic photosystems as both contain structurally and phylogenetically similar reaction centers and light-harvesting antenna complexes (Yurkov and Csotonyi, 2009).
2. In *Bacteroidetes* sp. HLUCCA01, *Rhodobacteraceae* sp. HLUCCA09, and *Erythrobacteraceae* sp. HL-111, the putative rhodopsin contains the RYXN(X_10_)Q transport motif characteristic of the NQ family of rhodopsins rather than the RYXD(X_10_)E proton-transport motif; NQ rhodopsins are common in hypersaline environments (Kwon *et al.*, 2013) and have recently been shown to transport sodium ions (Balashov *et al.*, 2014). This suggests that rhodopsins may perform other functions besides maintaining proton-motive force for ATP generation in these organisms, such as regulating osmotic pressure or driving efflux pumps via cation antiport (Fuhrman *et al.*, 2008).
3. RuBisCo catalyzes the addition of carbon dioxide to ribulose-1,5-bisphosphate and is required for the Calvin-Benson-Bassham reductive pentose phosphate cycle of carbon fixation (Tabita *et al.*, 2007; Erb *et al.*, 2012). Although *Bacteroidetes* sp. HLUCCA01 also contains an *rbcL* homolog, its catalytic motif is similar to the form IV RbcLs of *Rhodopseudomonas palustris* (gi: 77687805) and *Rhodospirillum rubrum* (gi: 48764419) as it is ~100 residues shorter than form I-III RbcLs and His replaces the canonical Glu^204^ residue in the catalytic motif (Carré-Mlouka *et al.*, 2006). Form IV Rubiscos, also termed RuBisCo-like proteins, have been found to lack the ability to fix carbon dioxide but instead catalyze an enolization reaction important for the salvage of methionine from methylthioadenosine (Tabita *et al.*, 2007; Erb *et al.*, 2012). Consequently, this gene is unlikely to permit autotrophy in *Bacteroidetes* sp. Bin01. HLUCCA01.
4. *Roseibaca calidilacus* HL-91, *Marinobacter* sp. HL-58, *Marinobacter excellens* HL-55, and *Algoriphagus marincola* HL-49 encode thioredoxin-dependent adenosine 5’-phosophosulfate (APS) reductases (TIGR2055 family); these enzymes are capable of reducing both APS and PAPS to sulfite.
